# Supplementary material for: How to Choose? Comparing Different Methods to Count Wolf Packs in a Protected Area of the Northern Apennines
Source: Genes (Basel). 2023 Apr 18;14(4):932. doi: 10.3390/genes14040932 (PMC10137897; doi:10.3390/genes14040932)
Supplement: Supplementary file 1 [file genes-14-00932-s001.zip › genes-2326935-supplementary.pdf]

Supplementary Material

File S1

Laboratory methods, population structure and ethical statements

1. We identified individual genotypes for samples at 12 unlinked autosomal canine microsatellites (short tandem repeats [STR]): 7 dinucleotides (CPH2, CPH4, CPH5, CPH8, CPH12, C09.250, and C20.253) and 5 tetranucleotides (FH2004, FH2079, FH2088, FH2096, and FH2137), selected for their high polymorphism and reliable scorability for wolves and dogs. We determined sex of samples using a polymerase chain reaction (PCR)–restriction fragment length polymorphism assay of diagnostic ZFX/ZFY gene sequences [45,57]. We used a panel of 6 STR to identify the genotypes with Hardy–Weinberg probability-of-identity (PID) among unrelated individuals, PID = 8.2×10^{-6}, and expected full siblings, $PID_{sibs} = 7.3 \times 10^{-3}$ [74,75] in the reference Italian wolves. We used another panel of 6 STR, also selected for their polymorphism and reliable scorability, to increase the power of admixture and kinship analyses, decreasing the PID values to PID = 7.7×10^{-9} and $PID_{sibs} = 3.1 \times 10^{-4}$. We identified maternal haplotypes by sequencing 350 base pairs of the mitochondrial DNA (mtDNA) control region, diagnostic for the haplotype W14, which is unique to the Italian wolf population, using primers L-pro and H350 [76]. We identified paternal haplotypes by typing 4 Y-linked microsatellites (Y-STR), MS34A, MS34B, MSY41A, and MS41B [43], characterized by distinct allele frequencies in dogs and wolves [77].

2. We amplified autosomal and Y-linked STR loci in 7 multiplexed primer mixes using the QIAGEN Multiplex PCR Kit (Qiagen Inc.), a GeneAmp PCR System 9700 Thermal Cycler (Applied Biosystems, Foster City, California), and the following thermal profile: 94°C for 15 min, 94°C for 30 s, 57°C for 90 s, 72°C for 60 s (40 cycles for scat, urine, and hair samples, and 35 cycles for muscle and blood samples), followed by a final extension step of 72°C for 10 min. We carried out amplifications in 10-μl volumes including 2 μl of DNA extraction solutions from scat, urine, and hair samples, 1 μl from muscle or blood samples (corresponding to approximately 20–40 ng of DNA), 5 μl of QIAGEN Multiplex PCR Kit, 1 μl of QIAGEN Q solution (Qiagen Inc.), 0.4 μM deoxynucleotide triphosphates, from 0.1 to 0.4 μl of 10 μM primer mix (forward and reverse), and RNase-free water up to the final volume. We amplified the mtDNA control region in a 10-μl PCR, including 1 or 2 μl of DNA solution, 0.3 pmol of the primers L-Pro and H350, using the following thermal profile: 94°C for 2 min, 94°C for 15 s, 55°C for 15 s, 72°C for 30 s (40 cycles), followed by a final extension of 72°C for 5 min. PCR products were purified using exonuclease/shrimp alkaline phosphatase (Exo-Sap; Amersham, Freiburg, Germany) and sequenced in both directions using the Applied Biosystems Big Dye Terminator kit (Applied Biosystems, Foster City, California) with the following steps: 96°C for 10 s, 55°C for 5 s, and 60°C for 4 min of final extension (25 cycles). DNA from scat, urine, and hair samples was extracted, amplified, and genotyped in separate rooms reserved to low template DNA samples, under sterile ultraviolet laminar flow hoods, following a multiple-tube protocol [45,57], including both negative and positive controls. We obtained genotypes from blood and muscle DNA, replicating the analyses twice. DNA sequences and microsatellites were analyzed in a 3130XL ABI automated sequencer (Applied Biosystems), using the ABI software SEQSCAPE 2.5 for sequences, and GENEMAPPER 4.0 for microsatellites (Applied Biosystems).

3. We assigned individual genotypes to their population of origin (wolves or dogs) using STRUCTURE 2.3 [47]. We ran STRUCTURE with 5 replicates of 10^4 burn-in followed by 10^5 iterations of the Markov chain Monte Carlo sampling, selecting the ''admixture'' model (each individual may have ancestry in more than 1 parental population), either assuming independent or correlated allele frequencies. We identified the optimal number of populations K using the ΔK procedure [78]. At the optimal K we assessed the average proportion of membership (Q_i) of the sampled populations (wolves or dogs) to the inferred clusters. We assigned genotypes to the Italian wolf or dog clusters at threshold q_i = 95 (individual proportion of membership [79]), or identified them as admixed if their qi values were intermediate. We checked putative wolf x dog hybrids further using additional admixture analyses on observed and simulated genotypes obtained by HYBRIDLAB [80] and using diagnostic mtDNA and Y-STR haplotypes.

4. No ethics permit was required for this study and no animal research ethics committee prospectively was needed to approve this research or grant a formal waiver of ethics approval since the collection of wolf samples involved dead animals. Fieldwork procedures were specifically approved by ISPRA as a part of national wolf monitoring activities. Dog blood samples were collected by veterinarians during health examinations with a not-written (verbal) consent of their owners (students/National park volunteers/or specialized technician personnel of the Italian Forestry Authority (CFS)), since they were interested on wolf conservation studies and monitoring projects in Italy. More over there is not a relevant local law/legislation that exempts our study from this requirement. Additionally, no anesthesia, euthanasia, or any kind of animal sacrifice was applied for this study and all blood samples were obtained aiming at minimizing the animal suffering.

5. Reference wild parentals were selected from found-dead wolves collected across the Italian peninsular distribution range that showed the typical wild coat color pattern and no other apparent dog-like traits such as white claws or spurs on the hind legs. Reference domestic parentals were selected from free-ranging mongrels and village dogs sampled in the same areas of the reference wolves, plus one male and one female randomly chosen from 14 wolf-sized dog breeds. Given the high between-breed variation these samples could represent a good proxy of the diversity in dogs while avoiding significant sub-structuring during clustering analyses. As wild and domestic reference individuals, all available in the ISPRA canid database, we only retained those whose genotypes showed no missing data and proportions of membership q_i>0.990 to the respective wild or domestic clusters estimated in previous Bayesian assignment procedures performed, using the software STRUCTURE 2.3, on 39 canine STRs commonly used to reconstruct individual genotypes in some of the most recent studies on wolf x dog hybridization in Europe. This conservative q-threshold was selected to avoid the inclusion of older admixed individuals among the wild reference population, thus reducing the power to correctly identify admixed individuals in the tested dataset. Furthermore, 90 of the selected reference wolves and 30 of the selected reference dogs were also tested in Maximum-Likelihood assignment procedures performed analyzing 156K genome-wide canine SNPs in the software Admixture v.1.2395 and confirmed their pure status showing q_i>0.990.

Costs and working time

Table S1. Total equipment cost for each technique.

	Item description	Price/unit (€)	Quantity	Total (€)	Total technique (€)
WH	Megaphones	35.24	12	422.88	720.96
	SD cards 16 GB	9.84	12	118.08	
	Compasses	15.00	12	180.00	
CT	Camera traps	168.90	39	6,587.10	12,168.80
	Camera trap security boxes	34.16	15	512.40	
	Python cables	26.70	39	1,041.30	
	SD cards 32 GB	28.00	78	2,184.00	
	AA batteries in packs of 10	4.50	39	175.50	
	6V batteries + connection jack	18.00	78	1,404.00	
	6V battery chargers	14.75	10	147.50	
	Padlocks	3.00	39	117.00	
NGS	Sampling material & genotyping	100.00	294	29,400.00	29,400.00
